# Relationships to land as a determinant of wellness for Indigenous women, two-spirit, trans, and gender diverse people of reproductive age in Toronto, Canada

**DOI:** 10.17269/s41997-022-00678-w

**Published:** 2022-08-30

**Authors:** Danette Jubinville, Janet Smylie, Sara Wolfe, Cheryllee Bourgeois, Nicole S. Berry, Michael Rotondi, Kristen O’Brien, Scott Venners

**Affiliations:** 1 Faculty of Health Sciences, Simon Fraser University, Burnaby, British Columbia Canada; 2 Ekw’í7tl Indigenous Doula Collective, Vancouver, British Columbia Canada; 3 Centre for Urban Health Solutions, Li Ka Shing Knowledge Institute, St. Michael’s Hospital, Toronto, Ontario Canada; 4 Dalla Lana School of Public Health, University of Toronto, Toronto, Ontario Canada; 5 Seventh Generation Midwives Toronto, Toronto, Ontario Canada; 6 School of Kinesiology and Health Science, York University, Toronto, Ontario Canada

**Keywords:** Canada, Health services, Health equity, Indigenous, Reproductive health, Reproductive justice, Canada, services de santé, équité en santé, autochtones, santé reproductive, justice reproductive

## Abstract

**Objective:**

Disparities in Indigenous reproductive health reflect Canada’s historic and ongoing colonial relationship with Indigenous peoples, which includes persistent inequities in health and social services. Reproductive justice scholars and activists advocate for intersectional approaches to enhancing Indigenous health equity that recognize land as a central determinant of wellness. The purpose of this study is to examine the association between relationships to land and wellness in a study of urban Indigenous women, two-spirit, trans, and gender diverse people of reproductive age in Canada’s largest city, Toronto.

**Methods:**

Data were obtained from the cross-sectional Our Health Counts (OHC) Toronto study, which employed respondent-driven sampling methods (*n* = 323) and a community-directed comprehensive health assessment survey. In an exploratory analysis, we took an Indigenous reproductive justice theoretical approach to multivariable logistic regression.

**Results:**

After adjusting for covariates, there was a statistically significant positive association between relationships to the land and wellness that was estimated with good precision (OR 3.7, 95% CI 2.5–5.3).

**Conclusion:**

Our findings indicate that among urban Indigenous women, two-spirit, trans, and gender diverse people of reproductive age there is a positive association between feeling strong in their relationships to land and feeling balanced in the four domains of health (physical, spiritual, mental, and emotional). The community-based, community-directed design of OHC Toronto was congruent with a reproductive justice approach to research. Reproductive justice theories are adaptable to quantitative research on Indigenous reproductive health and can yield novel insights for supporting Indigenous wellness.

**Supplementary Information:**

The online version contains supplementary material available at 10.17269/s41997-022-00678-w.

## Résumé

**Objectif:**

Les disparités que connaissent les peuples autochtones sur le plan de la santé reproductive sont le reflet des relations coloniales historiques et continues du Canada avec ces peuples, encore entachées d’iniquités dans les domaines de la santé et des services sociaux. Théoricien·nes et militant·es de la justice reproductive préconisent des approches intersectionnelles pour améliorer l’équité en santé chez les peuples autochtones, approches qui reconnaissent le territoire comme un déterminant central du bien-être. Nous avons voulu examiner l’association entre les relations avec le territoire et le bien-être dans une étude menée auprès de femmes et de personnes autochtones bispirituelles, trans et de diverses identités de genre en âge de procréer vivant à Toronto, la plus grande ville du Canada.

**Méthode:**

Nos données proviennent de l’étude transversale Our Health Counts (OHC) Toronto, qui a employé des méthodes d’échantillonnage en fonction des répondant·es (*n* = 323) et une enquête d’évaluation globale de la santé dirigée par la communauté. Dans une analyse exploratoire, nous avons employé la régression logistique multivariée selon une approche théorique de justice reproductive autochtone.

**Résultats:**

Après l’apport d’ajustements pour tenir compte des covariables, nous avons observé une association positive significative entre les relations avec le territoire et le bien-être, que nous avons estimée avec une bonne précision (RC 3,7, IC de 95 % 2,5–5,3).

**Conclusion:**

D’après nos constatations, il y a chez les femmes et les personnes autochtones bispirituelles, trans et de diverses identités de genre en âge de procréer vivant en milieu urbain une association positive entre un fort sentiment d’appartenance au territoire et un sentiment d’équilibre entre les quatre domaines de la santé (physique, spirituel, mental et émotionnel). L’étude OHC Toronto ayant été conçue par et pour la communauté, elle est conforme à une approche de recherche axée sur la justice reproductive. Les théories de la justice reproductive peuvent être adaptées à la recherche quantitative sur la santé reproductive autochtone, et elles peuvent jeter un nouvel éclairage sur les moyens d’appuyer le bien-être des personnes autochtones.

## Introduction

Reproductive well-being—which the World Health Organization (World Health Organization, 2019) defines as “a state of complete physical, mental, and social well-being and not merely the absence of disease or infirmity, in all matters relating to the reproductive system and to its functions and processes”—is essential to overall health in any society. In Canada, Indigenous (First Nations, Inuit, and Métis) people face intersecting inequities across determinants of health commonly associated with reproductive health, such as gendered violence and access to reproductive, sexual, and maternal healthcare (Nelson, 2017; Yee et al., 2011). Indigenous reproductive health inequities stem from colonial policies and processes designed to eliminate Indigenous people and gain access to Indigenous lands and resources (Gurr, 2015; Smith, 2005; Wolfe, 2006). Childbearing Indigenous people have endured state processes that specifically endangered Indigenous reproduction, such as the outlawing of Indigenous midwifery, child apprehension, routine evacuation for childbirth, coercive sterilization, and abusive abortions (Stote, 2015). Indigenous feminist scholars contend that colonial systems have targeted women and childbearing people because they hold representational significance as the ones who birth and raise Indigenous nations (Anderson, 2004; Million, 2013; Stote, 2015).

To address systemic inequities that impact Indigenous reproductive well-being, scholars and activists have been engaging the theoretical lens of reproductive justice since it was first introduced by Black feminists in 1994 (Ross and Solinger, 2017). Reproductive justice theorists take an intersectional, social justice–oriented approach to reproductive health equity, affirming the right of childbearing people to have (or not have) children, to maintain bodily autonomy, to gender and sexual freedom, and to parent children in safe and sustainable communities (Ross and Solinger, 2017). While not all Indigenous women, two-spirit, trans, and gender diverse people can, will, or want to have children, these groups have unique and specific reproductive health needs, and are disproportionately negatively impacted when these needs go unmet. By centring the knowledge and experiences of Indigenous childbearing people, Indigenous reproductive justice theories reveal the gendered, racialized, and sexualized nature of colonialism, and underscore the importance of Indigenous self-determination, land, and culture to reproductive well-being (Gurr, 2015).

Reproductive justice research has shown how Indigenous peoples’ relationships to land have resulted in disproportionate risks of reproductive harm, in particular through exploring the impacts of resource extraction and other pollution-producing projects on fetal development, breast/chestfeeding, birth rates, and gender-based violence in Indigenous communities (Hoover et al., 2012; Smith, 2005; Wiebe, 2016). Gendered colonial policies such as the Indian Act, residential schools, and child welfare systems deliberately undermined the relationships of Indigenous women, two-spirit, gender diverse, and LGBTQQI people to land (Million, 2013). Threats to the land uniquely impact childbearing people: degradation of ecosystems poses heightened health risks for childbearing people and infants, and development and resource extraction projects create known risks of gendered violence for Indigenous communities (Smith, 2005).

Land is particularly important to Indigenous reproductive justice because land is a foundational determinant of Indigenous health. Richmond (2015, p. 58) writes: “The relationship between First Nations peoples and the land is a multifaceted one, and formative for countless social determinants of health, including social relationships, spirituality, and access to foods and medicines.” Relationships with land enhance Indigenous wellness through facilitating cultural connection, positive identity formation, and health-promoting land-based lifeways, such as traditional food and medicine gathering (Parlee et al., 2005). Given the many barriers Indigenous childbearing people face to achieving well-being in a colonial context, scholars and activists theorize relationships to land as crucial to reproductive justice (Danforth, 2010; Wiebe, 2016).

Despite what is known about the significance of land to Indigenous health, very little quantitative research explores the connection between relationships to land and *wellness* among the childbearing population. This is particularly true for urban centres. The majority of Indigenous people in Canada live in cities (Statistics Canada, 2017), which are located on Indigenous lands. Without quantitative data to influence health policy and practice by demonstrating the significance of land to the wellness of Indigenous childbearing people, research gaps in this area create potential barriers to Indigenous reproductive justice.

The purpose of this paper is to examine whether relationships to land are associated with wellness in a sample of urban Indigenous women, two-spirit, trans, and gender diverse people of reproductive age (aged 15–44) living in Toronto, Ontario. This research question was determined through a community-based research partnership with two Indigenous-led organizations, the Seventh Generation Midwives Toronto (SGMT) and the Well Living House Action Research Centre for Indigenous Infant, Child and Family Health and Well-Being (WLH), which is based at St. Michael’s Hospital (SMH), Unity Health Toronto. Data came from Our Health Counts (OHC) Toronto, an urban Indigenous population health study led by SGMT and WLH. In an exploratory effort, this secondary analysis took an Indigenous reproductive justice theoretical approach to multivariable regression. Regression modelling was chosen for its compatibility with the OHC Toronto data collection method, respondent-driven sampling (RDS).

## Methods

### Our Health Counts Toronto

Located in the traditional territories of the Mississaugas of the Credit, the Anishnabeg, the Chippewa, the Haudenosaunee, and the Wendat peoples, Toronto is Canada’s largest metropolis. Approximately 55,000 First Nations, Inuit, and Métis people from diverse nations live in the census metropolitan area (Rotondi et al., 2017). As in other Canadian cities, limitations with national statistics have resulted in significant data gaps concerning the health of urban Indigenous people in Toronto (Rotondi et al., 2017). These data gaps have impacted the quality and availability of services that promote reproductive well-being for urban Indigenous peoples.

To develop a comprehensive health database by and for Indigenous peoples living in the City of Toronto, the OHC Toronto study was conducted by WLH and SGMT between 2014 and 2018. Indigenous values, data sovereignty principles of Ownership, Control, Access, and Possession (OCAP©), and SGMT ownership and control of data holdings are embedded in a research, publication, and data-sharing agreement between SGMT and the WLH. The community-partnered, community-directed governance structure of OHC Toronto includes an Advisory Council of Indigenous Grandparents as well as collaborative involvement of over 20 Indigenous and allied service providers who co-developed the survey. SGMT acts as the data custodian, responsible for ownership and control of data holdings.

OHC Toronto was given ethics approval by the SMH Research Ethics Board (REB# 14-083c). As a secondary analysis, this offshoot study was conducted through a community-based research partnership governed by the OHC Toronto Data Use Protocol Agreement. This study was also given ethical approval by the Office of Research Ethics at Simon Fraser University (REB# 2018s0180).

### Reproductive justice approach

OHC Toronto partners and collaborators identified reproductive health as a research priority, given the importance of reproductive health measures to overall population health (Wolfe et al., 2018). Survey data showed that the fertility rate for Indigenous people of reproductive age is 2.12 children, compared to 1.51 per woman living in Ontario (Wolfe et al., 2018). Based on OHC Toronto’s population size estimate, approximately 1036–1408 children are expected to be born to Indigenous women, two-spirit, trans, and gender diverse people per year in the City of Toronto (Wolfe et al., 2018). While there is demonstrated need for reproductive health services, 27% of Indigenous adults in Toronto believe reproductive health services are inadequate (Wolfe et al., 2018).

This study arises from a community-based research partnership between WLH, SGMT, and co-author Jubinville, a Cree/Saulteaux/Jewish/European woman who is a Vancouver-based graduate student, mother, and doula. These partners share a vision for enhancing equity in reproductive health services for Indigenous peoples, and this vision was brought forward into our study approach. Given the lack of studies that apply an Indigenous reproductive justice theoretical lens to statistical analyses, our efforts to do so are exploratory and rely on several methodological choices modelled after common practices in the existing literature (see Hoover et al., 2012; Gurr, 2015; Wiebe, 2016).

The methodological choices that form the basis of our reproductive justice approach include the following: (1) research is conducted through a community-led research partnership; (2) research is contextualized and analyzed drawing on Indigenous and intersectional feminist perspectives on reproductive justice; (3) researchers offer a positionality statement; (4) research draws on Indigenous knowledge for understanding reproductive health; (5) research methods seek to be inclusive and gender-affirming; (6) strengths-based, community-led solutions are highlighted where possible; and (7) the purpose of the research is to advance Indigenous peoples’ goals for reproductive justice. We expand below on how these choices informed our statistical model.

### OHC sampling strategy

Indigenous community surveyors who received specialized training in cultural safety and sensitivity collected OHC Toronto’s baseline health data between April 13, 2015 and March 31, 2016, using respondent-driven sampling. RDS aims to capture hard-to-reach populations by tapping into social networks, similar to chain referral sampling (Heckathorn, 1997). The initial sample included 916 adults aged 15+ who self-identified as Indigenous (First Nations, Inuit, and/or Métis) and lived or accessed health and/or social services in the City of Toronto. This included the exception of one non-Indigenous respondent who had Indigenous children. Sampling for OHC Toronto was reported previously (Rotondi et al., 2017).

### Indigenous reproductive health sample

Following other OHC Toronto reproductive health analyses (Wolfe et al., 2018), the selection criteria for our sample included those who: (1) participated in the OHC Toronto Adult Survey; (2) identified as a woman, trans, or gender diverse person (this included people who further identified as two-spirit, which was asked as a separate question); and (3) were aged between 15 and 44 inclusive at the time of the survey (*n* = 323). All OHC Toronto Adult Survey participants who identified as men or were over the age of 45 were excluded.

### Directed acyclic graph

**Fig. 1:**
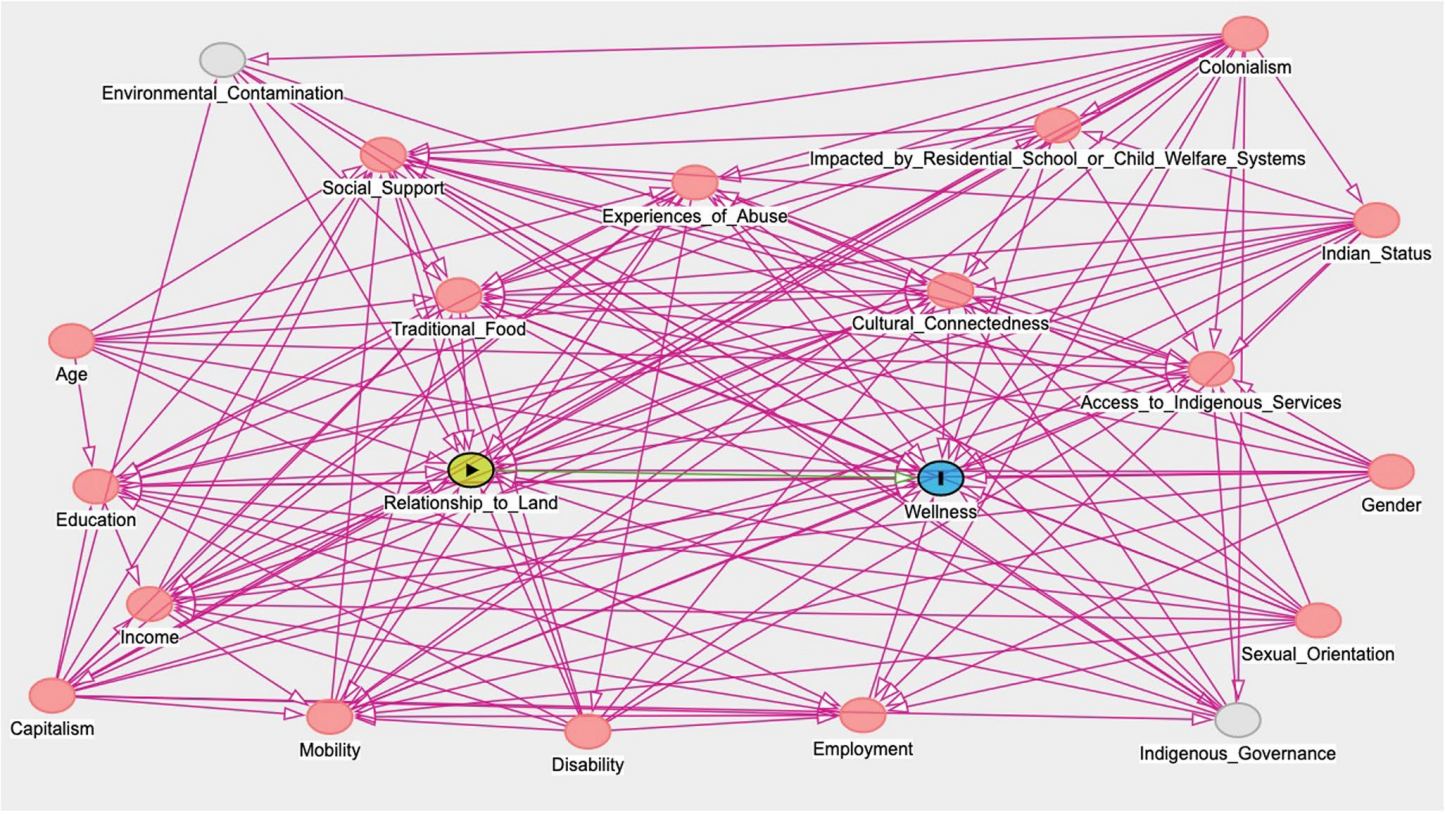
Directed acyclic graph (DAG) of effect of relationship to land on wellness

Directed acyclic graphs (DAGs) are commonly used in health research when estimating causal effects (Tennant et al., 2021). DAGs help researchers to identify which variables require conditioning for control of confounding, while also providing “a simple and transparent way for observational data scientists to identify and demonstrate their knowledge, theories and assumptions about the causal relationships between variables” (Tennant et al., 2021, p. 622). Our DAG (Figure 1) was designed by Jubinville and reflects two main sources of subject matter knowledge: first, the knowledge, theories, and assumptions gleaned from the reproductive justice and Indigenous feminist literature; second, Jubinville’s background causal knowledge stemming from her own experiences as an urban Indigenous woman and birthworker as well as conversations with the OHC Toronto community partners and collaborators.

### Variables

By following the graphical rules for completing a DAG (Greenland and Robins, 1999), we identified 19 covariates that required conditioning for control of confounding in the association between our exposure variable “relationship to land” and the outcome “wellness.” Fifteen covariates were measured using data from the OHC Toronto survey and two were unmeasured: “environmental contamination” and “Indigenous governance.” Two covariates (“colonialism” and “capitalism”) were not included in the model but were considered controlled for as everyone in the target population was assumed to have the same value for these macro variables.

For analysis purposes, the outcome variable “wellness” was recoded as a binary variable, with one category representing those who reported feeling balanced in their physical, emotional, mental, and spiritual health all or most of the time. This construct of wellness was determined through OHC Toronto’s community-partnered survey development and reflects the holistic concept of balance between the four domains of health, often expressed in Indigenous health spaces through referencing the Medicine Wheel (Quinless, 2022).

The exposure variable, “relationship to land,” was also recoded into a dichotomous variable, with one category representing those who reported feeling strong in their relationship to the land all or most of the time. To enhance power and precision in the model, data for measuring covariates were collapsed into dichotomous categories, with the exception of the continuous variable, age. (For variable definitions, see Tables 1-2 in Supplementary Material.)

**Table 1:** Distributions of variables by wellness among women, two-spirit, trans, and gender diverse people of reproductive age (*n* = 308) in the “Our Health Counts Toronto” survey. Toronto, Canada, 2015–2016

	Wellness	
	Feels balanced all or most of the time	
	No (*n = 193)*	Yes (*n = 115)*
	*n* (%)	*n* (%)
Feels strong in relationship to land		
All or most of the time	87 (45.1)	81 (70.4)
Some, a little, or none of the time	106 (54.9)	34 (29.6)
Accessed Indigenous services in past year		
Yes	178 (92.2)	111 (96.5)
No	15 (7.8)	4 (3.5)
Age (average)	34.4	34.4
Sense of cultural connectedness		
Strong	151 (78.2)	103 (89.6)
Low	42 (21.8)	12 (10.4)
Health impacted by a physical or mental disability		
Yes	101 (52.3)	55 (47.8)
No	92 (47.7)	60 (52.2)
Level of education		
High school or more	125 (64.8)	75 (65.2)
Did not complete high school	68 (35.2)	40 (34.8)
Employment status		
Employed in some capacity or n/a	104 (53.9)	57 (49.6)
Unemployed	89 (46.1)	58 (50.4)
Experiences of physical, mental, or sexual abuse		
Yes	104 (53.9)	57 (49.6)
No	89 (46.1)	58 (50.4)
Gender		
Cisgender	160 (82.9)	87 (75.7)
Two-spirit, trans, or gender diverse	33 (17.1)	28 (24.3)
Feels health impacted by residential school or child welfare systems		
Yes	110 (57.0)	64 (55.7)
No	83 (43.0)	51 (44.3)
Income		
Below low-income cut-off	150 (77.7)	97 (84.3)
Above low-income cut-off	43 (22.3)	18 (15.7)
Indian status		
Non-status	125 (64.8)	82 (71.3)
Status	68 (35.2)	33 (28.7)
Moved in the past year		
No	113 (58.5)	74 (64.3
Yes, at least once	80 (41.5)	41 (35.7)
Sexual orientation		
Heterosexual	159 (82.4)	84 (73.0)
Other sexual orientations	34 (17.6)	31 (27.0)
Has warm and trusting relationships with others		
Never or less than every day	120 (62.2)	48 (41.7)
Every day	73 (37.8)	67 (58.3)
Eats traditional foods		
A few times or not at all in the past year	171 (88.6)	99 (86.1)
Often in the past year	22 (11.4)	16 (13.9)

**Table 2:** Relative odds of wellness by relationship to land among women, two-spirit, trans, and gender diverse people of reproductive age (*n* = 308) in the “Our Health Counts Toronto” survey. Toronto, Canada, 2015–2016

Variable	Crude		Adjusted*	
	OR	95% CI	OR	95% CI
Relationship to land	3.6	1.2 to 10.6	3.7	2.5 to 5.3
Access to Indigenous services			10.9	4.3 to 27.3
Age			1.0	0.9 to 1.0
Cultural connectedness			0.9	0.4 to 2.1
Disability			2.6	1.7 to 3.9
Education			1.3	0.7 to 2.3
Employment			1.1	0.3 to 3.4
Experiences of abuse			1.2	0.7 to 2.2
Gender			0.4	0.1 to 2.6
Impacted by residential school or child welfare policies			0.8	0.4 to 1.6
Income			0.5	0.0 to 5.2
Indian status			0.5	0.2 to 1.3
Mobility			3.4	2.5 to 4.7
Sexual orientation			0.7	0.3 to 2.1
Social support			2.2	0.7 to 7.5
Traditional foods			9.0	1.0 to 78.4

*Associations between covariates and the outcome should not be interpreted as total causal effects

### Statistical analysis

Logistic regression modelling within the potential outcomes framework of causal modeling was used to estimate the total causal effect of relationships to land on wellness. The GLIMMIX procedure in SAS® V.9.4 was used to estimate weighted and adjusted associations between measurements, following conclusions of previous OHC studies that tested various modelling approaches (Beckett et al., 2018; Kitching et al., 2019). Models were weighted to account for unequal probability of sampling due to variability in individual network size, as reported previously (Beckett et al., 2018).

Odds ratios (ORs) and 95% confidence intervals (CIs) were generated for the total effect of relationship to land on wellness. Statistical significance was judged at alpha=0.05 (two-sided). Data were missing from 8 variables; these 15 missing cases were handled by case deletion (*n* = 308).

## Results

Table 1 shows the distribution of variables by wellness. Table 2 shows that after adjusting for all 15 measured covariates there was a statistically significant positive association between wellness and relationship to land that was estimated with good precision (OR 3.7, 95% CI 2.5–5.3).

## Discussion

This study hypothesized that relationships to land would have a positive association with wellness. After adjusting for covariates, we found a statistically significant and meaningful effect (3.7, 95% CI 2.5–5.3). Our findings indicate that among urban Indigenous women, two-spirit, trans, and gender diverse people of reproductive age there is a positive association between feeling strong in their relationships to land and feeling balanced in their physical, spiritual, mental, and emotional health. Reproductive justice theorists argue relationships to land are foundational to the wellness of childbearing Indigenous people; these findings add quantitative evidence to support this argument.

Advancing reproductive justice for Indigenous peoples requires mobilization around relational, land-based concepts of wellness and care in reproductive health services. Indigenous models of reproductive care, such as Indigenous midwifery, affirm the importance of land to the wellness of childbearing people through honouring language, traditional medicines, oral traditions, ceremonies, kinship, and Indigenous values in service delivery (National Aboriginal Council of Midwives, 2016). The findings of OHC Toronto confirm the success of midwifery services for the urban Indigenous population of Toronto; 32% of Indigenous people used a midwife as their prenatal care provider, compared to 8% of women in the Toronto Central Local Health Integration Network (Wolfe et al., 2018).

Reproductive events such as birth, menarche, pregnancy, postpartum, menopause, and death are significant opportunities for engaging cultural practices that strengthen relationships to land. Historically, Indigenous birthworkers tended to these transitions as rites of passage through providing land-based medical and spiritual care, but today are constrained by jurisdictional issues, health policies, educational barriers, and funding challenges (National Aboriginal Council of Midwives, 2016). The Indigenous-led movement for “Birth on Country” in urban and rural areas of Australia highlights the need for increased access to Indigenous birthworkers, alongside other strategies that strengthen birthers’ and infants’ relationships to land, such as birthing close to home, extended family support, and placenta ceremonies (Marriott et al., 2019).

This study fits within a growing body of literature related to Indigenous reproductive justice. Indigenous reproductive justice research calls for transformation to health policies and services to reflect Indigenous intersectional, decolonial, and socioecological philosophies on health and wellness. In addition to culturally relevant and culturally safe reproductive health services, this transformation may include broader implementation of the United Nations Declaration on the Rights of Indigenous Peoples and the calls to action from the National Inquiry into Missing and Murdered Indigenous Women and Girls. Enacting the principles of reproductive justice may also include reform to policies and practices that are known to cause harm to Indigenous people and disrupt Indigenous relationships to land, such as the routine evacuation of pregnant Indigenous women from rural and remote communities, child apprehensions, resource extraction, and other development projects that put the environment at risk.

### Strengths and limitations

A strength of this study was the community-based partnership with OHC Toronto, which gathered comprehensive population-level demographic, health status, and health service use information for Indigenous peoples in Toronto through exercising principles of OCAP®. Particular strengths of OHC Toronto were the community-partnered survey design and sampling strategy, which engaged Indigenous subpopulations that have historically been marginalized in national statistics, such as two-spirit, trans, and gender diverse Indigenous people. The community-directed, Indigenous-led nature of OHC Toronto resulted in an ideal dataset for exploring the possibility of approaching logistic regression through the lens of reproductive justice.

The inclusive survey design of OHC Toronto offered participants multiple categories for self-identification of Indigenous heritage, gender, and sexuality. While this was a strength of the study, low sample sizes of trans and gender diverse participants precluded the possibility of disaggregating data without compromising participant safety and confidentiality. Kitching et al. (2019) note that the RDS design of OHC Toronto may have also undersampled Métis and Inuit subpopulations. Because the experiences and perspectives of historically marginalized community members are critical to Indigenous reproductive justice, future studies could consider oversampling these populations or using mixed methods to enrich the data.

The exposure and outcome variables in this study (relationship to land and wellness) relied on self-reports. While self-reported measures respect the autonomy of individuals in determining their own health status, the data may be subject to recall error. Attempts to reduce recall error were made through community-based survey development and the appointment of Indigenous community surveyors with cultural safety and sensitivity training. This study was also limited by the cross-sectional nature of the data because we could not discount that part of the association we observed was due to the reverse effect of wellness on relationship to land. Future studies could better observe dynamic concepts such as wellness and relationship to land through a longitudinal design.

In this study, the outcome variable “wellness” was constructed based on a holistic concept of health and determined through OHC Toronto’s community-partnered survey development. Whether or not this construct of wellness resonates for all OHC Toronto participants is beyond the scope of this paper. As Indigenous health research trends towards locally grounded, Nation-specific knowledge and concepts, researchers must collaborate with communities to determine which constructs of wellness are valid within their own knowledge systems and how best to measure them.

Reliance on secondary data created inherent limitations with missing or imperfectly constructed data for concepts in our DAG. Considering that this study was designed after OHC data collection was complete, it is a testament to the success of the community survey design that data were available to measure most variables prescribed by our DAG. Of 19 covariates required to control for confounding, only two could not be included in our model: “environmental contamination” and “Indigenous governance.” Confounding was partially addressed by controlling for mediators between missing covariates and “relationship to land” and “wellness.” Nonetheless, missing data and potential construct invalidity may have led to some residual confounding in the model. This potential confounding, if present, would most likely lead to a bias away from the null.

We used a DAG to illustrate our way of thinking about the causal relationships that confound the association between relationships to land and wellness. Too often, assumptions made by quantitative researchers lack transparency and/or reflect colonial worldviews (Walter and Andersen, 2013). We aimed to avoid these pitfalls by making our concept of reality explicit through the use of a DAG. It is possible that other researchers might generate a different DAG for the effect of relationship to land on wellness based on their own subject matter knowledge (Hernán et al., 2002).

By using a DAG, we were able to extend our Indigenous reproductive justice theoretical approach into our statistical model. Graphical rules about how to complete the DAG resulted in a complex map of covariates that confound the causal connection between relationships to land and wellness. Data collected using RDS methods require large sample size due to large design effects, and the high number of covariates exacerbated existing challenges with power and precision in our model. Researchers interested in engaging these methods should note the requirement for large sample sizes. We also note that RDS methodological development is ongoing. While we used weighted models for this study, emerging evidence suggests that unweighted models may be appropriate for RDS regression analyses (Avery et al., 2019).

## Conclusion

The community-partnered, community-directed methodology of OHC Toronto facilitated a strengths-based exploration of the association between relationships to land and wellness among urban Indigenous childbearing people and was congruent with a reproductive justice approach. Reproductive justice research emphasizes the ways in which Indigenous people and lands are intimately connected, and how policies and practices that disregard Indigenous peoples’ knowledge and agency over their own bodies and their own lands create reproductive harms that span generations. The findings of this study reflect community and research understandings that relationships to land are not only a risk factor for illness, they are also important to wellness.

This study adds quantitative evidence to the growing field of Indigenous reproductive justice research, which calls for investments into reproductive health services that strengthen Indigenous relationships to land, such as Indigenous midwifery care, and which troubles policies and practices that diminish Indigenous relationships to land, such as resource extraction, for being harmful to reproductive well-being. The exploratory design of this study found that reproductive justice theories were adaptable to quantitative research focused on Indigenous reproductive health equity.

### Contributions to knowledge

What does this study add to existing knowledge?

- Our exploratory study took an Indigenous reproductive justice theoretical approach to secondary statistical analysis.
- Our reproductive justice approach relied on several methodological choices aimed at centring Indigenous knowledge and community perspectives. This approach is extended to our statistical model, as illustrated by a directed acyclic graph (DAG).
- We found a statistically significant and positive association between relationships to land and self-reported wellness among urban Indigenous women, two-spirit, trans, and gender diverse people of reproductive age (15-44).
- Our findings add to academic and community understandings of land as a foundational determinant of Indigenous reproductive well-being.

What are the key implications for public health interventions, practice or policy?

- Our findings suggest that policies, practices, and interventions aimed at strengthening Indigenous relationships to land could have a positive impact on the health and well-being for urban Indigenous women, two-spirit, trans, and gender diverse people of childbearing age.
- In light of our findings, relational, land-based models of health and care can be seen as a best practice for enhancing Indigenous reproductive health services. Indigenous birthworkers, such as midwives and doulas, advance Indigenous reproductive justice and wellness by promoting connections to land through incorporating language, traditional foods and medicines, ceremonies, and Indigenous values into their services for childbearing people.

## Supplementary information

ESM 1 (PDF 83 kb)
