# Supplementary material for: Relationships to land as a determinant of wellness for Indigenous women, two-spirit, trans, and gender diverse people of reproductive age in Toronto, Canada
Source: Can J Public Health. 2022 Aug 30;115(Suppl 2):253–62. doi: 10.17269/s41997-022-00678-w (PMC11582287; doi:10.17269/s41997-022-00678-w)
Supplement: Supplementary file 1 — (PDF 83 kb) [file 41997_2022_678_MOESM1_ESM.pdf]

Supplementary Material

Table 1. Exposure and outcome variables from the Our Health Counts Toronto study survey

Variable	Question	Response Option	Survey Question
Wellness (outcome)	How often do you feel that you are in balance in the four aspects (e.g. physical, emotional, mental, spiritual) of your life?	All of the time<br>Most of the time<br>Some of the time<br>A little of the time<br>None of the time	Section 4.2
Relationship to land	How often do you feel strong in your relationship to the land/ Mother Earth?	All of the time<br>Most of the time<br>Some of the time<br>A little of the time<br>None of the time	Section 4.3

Table 2. Variables Constructed to Control for Confounding

Variable Name	Concept	How Variable was Constructed	Data Source
Access to Indigenous Services	Accessing programs and services targeted towards Indigenous populations	A dichotomous variable, with those who did not access any Indigenous programs and services in one group (0), and those who accessed 1 or more services in another (1)	OHC Toronto
Age	Numeric age	A continuous variable representing numeric age	OHC Toronto
Capitalism	Living within the economic system of capitalism	This variable was considered conditioned as capitalism is the dominant economic system in Toronto	n/a
Colonialism	Living within the governance system of colonialism	This variable was considered conditioned as colonialism is the dominant system of governance in Toronto and Canada, from an Indigenous reproductive justice theoretical lens	n/a

Variable Name	Concept	How Variable was Constructed	Data Source
Cultural Connectedness	Feeling of connectedness to an Indigenous cultural identity and community of belonging. Determined using OHC Toronto’s adapted version of the Multi-Ethnic Identity Measure (MEIM-R) for an Indigenous context	A binary variable based on the total MEIM score, with strong scores (2.9 – 4.0) in one category (1), and other scores (1.0 - 2.9) in another category (0).	OHC Toronto
Disability	Possessing a physical or mental disability that impacts one’s health	A dichotomous variable, with those who reported having a physical or mental disability impacting health for 6 months or longer in one group (0), and those who did not in another (1)	OHC Toronto
Education	Level of education achieved	A dichotomous variable, with those who did not complete high school in one group (0), and those who completed high school or more in another (1)	OHC Toronto
Employment	Employment status	A dichotomous variable, with unemployed in one group (0), and all other employment categories in another (1)	OHC Toronto
Environmental Contamination	Levels of contamination that impact the overall health of the land, including the soil, air, water, animal, and plant life	This variable was unmeasured	n/a

Variable Name	Concept	How Variable was Constructed	Data Source
Experiences of Abuse	Having experienced household violence	A dichotomous variable, with those who reported having been physically hurt, insulted or talked down to, threatened with harm, screamed or cursed at, had their actions restrictions, or non-consensual sex in one group (0), and those did not report having any of those experiences in another (1)	OHC Toronto
Gender	Gender identity	A dichotomous variable, with two-spirit women, trans, and gender diverse people in one category (0), and cisgender women in another (1)	OHC Toronto
Impacted by Residential School or Child Welfare Systems	Firsthand or intergenerational experiences with residential schools and child apprehension	A dichotomous variable, with those who reported feeling that their health had been negatively impacted by the residential school and child welfare policies in one group (0), and those who did not report feeling that way in another (1)	OHC Toronto
Income	Income	A dichotomous variable, with those living below the low-income cut-off in one group (0), and those living above the LICO in another (1)	OHC Toronto
Indian Status	Indian Status	A dichotomous variable, with those who had Indian status in one group (0), and those who did not in another (1)	OHC Toronto
Indigenous Governance	Living within systems of Indigenous governance, which are rooted in Indigenous knowledge, legal, and kinship systems	This variable was unmeasured	n/a

Variable Name	Concept	How Variable was Constructed	Data Source
Mobility	Recently moving residence	A dichotomous variable, with those who moved 1 or more times in the last 12 months in one group (0), and those who moved 0 times in the last 12 months in another (1)	OHC Toronto
Sexual Orientation	Sexual Orientation	A dichotomous variable, with people who did not identify as heterosexual in one group (0), and heterosexual identifying people in another (1)	OHC Toronto
Social Support	Feeling connected and supported by a network of people	A dichotomous variable, with those who reported having warm and trusting relationships with others almost every day, about 2 or 3 times a week, about once a week, once or twice, or never in one group (0) and those who reported having warm and trusting relationships with others every day in another (1)	OHC Toronto
Traditional Foods	Consumption of traditionally hunted/gathered/grown and/or country foods	A dichotomous variable, with those who reported eating traditional foods a few times or not at all in the last 12 months in one group (0), and those who reported eating traditional foods often in the last 12 months in another (1)	OHC Toronto
